# Self-reported poor sleep quality on the day of delivery is a potential risk factor for postpartum depression after cesarean delivery: a retrospective cohort study

**DOI:** 10.1186/s40981-025-00833-5

**Published:** 2025-12-29

**Authors:** Kaede Watanabe, Shohei Noguchi, Yuki Shiko, Daisuke Sakamaki, Yohei Kawasaki, Yusuke Mazda

**Affiliations:** 1 Department of Obstetric Anesthesiology, Center for Maternal-Fetal and Neonatal Medicine, Saitama Medical Center, Saitama Medical University, 1981 Kamoda, Kawagoe, Saitama 350-8550 Japan; 2 Department of Biostatistics, Graduate School of Medicine, Saitama Medical University, Moroyama, Japan; 3 Department of Anesthesiology, Jikei University School of Medicine, Tokyo, Japan

**Keywords:** Cesarean delivery, Postpartum depression, Sleep disturbance

## Abstract

**Background:**

Postpartum depression (PPD) affects a significant number of women. Sleep disturbances have been associated with PPD; however, it remains unclear whether immediate postpartum sleep disturbances are risk factors. We investigated the potential association between self-reported poor sleep quality on the first day after cesarean delivery and the development of PPD, measured using the Edinburgh Postnatal Depression Scale (EPDS) scores.

**Methods:**

We conducted a retrospective cohort study of women who underwent elective cesarean delivery under neuraxial anesthesia. Patients were asked about their sleep quality on the day after surgery and divided into Poor and Good Sleep groups. The EPDS scores were recorded at one month postpartum. The postpartum EPDS scores were compared between the two groups, and potential confounding factors were adjusted for using propensity score matching.

**Results:**

A total of 256 patients (55 and 201 in the Poor and Good Sleep groups, respectively) were included in the analysis. After propensity score matching, the postnatal EPDS score was significantly higher in the Poor Sleep group compared to that in the Good Sleep group (6.7 ± 5.2 vs 4.5 ± 4.3, *P* = 0.007). Maternal PPD (i.e., EPDS ≥ 9) was also significantly higher in the Poor Sleep group (31.5% vs 17.5%, *P* = 0.045, OR 2.17 [95%CI 1.01–4.67]). The Poor Sleep group had higher endocrine morbidity and a higher rate of multiple gestations.

**Conclusion:**

There is a strong correlation between self-reported poor sleep quality on the first day after cesarean delivery and the EPDS score one month after childbirth. Sleep disturbances on the day of delivery may be a potential risk factor for PPD.

**Supplementary Information:**

The online version contains supplementary material available at 10.1186/s40981-025-00833-5.

## Introduction

Postpartum depression (PPD), a major new-onset depression within 1 month of delivery, affects 17% of postnatal women globally [1]. PPD has been shown to have a detrimental impact on mothers, children, and their families, resulting in postpartum suicide, a leading cause of maternal death in developed countries [2]. Additionally, PPD is associated with negative mother–child relationships and leads to impaired development, poor cognitive functioning, behavioral inhibition, and emotional maladjustment in children [2]. Therefore, it is important to identify the risk factors and implement effective preventive measures.

The pathophysiology of PPD is complex, involving several potential risk factors, including psychosocial stressors, hormonal changes, and alterations in sleep patterns [2]. Many studies have shown a significant association between perinatal sleep disturbance and the development of PPD, underscoring the need to address sleep disturbance as a potential target for the prevention and treatment of PPD [3]. However, it remains unclear whether sleep disturbance immediately following childbirth is a risk factor for the onset of PPD. Identifying such an association can enable healthcare providers to target sleep disturbances during postnatal hospital stays as a modifiable risk factor, thereby reducing the incidence of PPD.

Therefore, this study aimed to investigate the hypothesis that sleep disturbances within the first day after cesarean delivery are related to scores on the Edinburgh Postnatal Depression Scale (EPDS), a tool for screening PPD, 1 month after delivery.

## Methods

The study protocol was approved by the Institutional Research Ethics Committee of Saitama Medical Center, Saitama Medical University (#2022–041). Owing to the retrospective nature of the study clinical data were extracted from medical records and anonymized before analysis. Although individual consent was not obtained, an opt-out process was implemented in accordance with institutional guidelines.

### Data collection and study setting

This retrospective observational study was conducted at a single tertiary perinatal center in Saitama, Japan. The inclusion criterion was elective cesarean deliveries under neuraxial anesthesia between March 1, 2021, and February 28, 2022. A retrospective review was conducted using electronic medical records. The exclusion criteria were emergent surgery, cesarean delivery under general anesthesia, intrauterine fetal demise, and incomplete essential data, specifically missing information from the 24-h postoperative period or the 1-month postpartum checkup.

### Exposure

The exposure of interest was the occurrence of self-reported poor sleep quality in the first 24 h after cesarean delivery. Participants were interviewed the morning after childbirth as part of a postoperative round by the obstetric anesthesia team. They were asked to report the quality of their sleep during the initial postoperative night using a standardized question: “Did you sleep well last night?”. The responses (Yes/No) were recorded immediately after the round. Subsequently, investigators reviewed the medical records. Based on their responses, participants were categorized into either a good or a poor postoperative sleep group. Those who answered 'Yes' were classified into the good sleep group, and those who answered 'No' were classified into the poor sleep group. We employed this binary, simple question as our aim was to establish an uncomplicated method for assessing sleep quality, sufficient for the preliminary screening of sleep disturbances.

### Outcomes

The primary outcome was the EPDS score recorded at the 1-month postpartum checkup. The EPDS is a widely recognized and validated tool for screening PPD. This questionnaire comprises 10 questions, and results are assessed by a total score ranging from 0 to 30, with lower scores indicating lower levels of depression [4]. The Japanese version of the EPDS has reliability and validity, with a cut-off score of ≥ 9, indicating a risk of maternal depression in the Japanese population [5].

### Anesthesia management during surgery

This study investigated the administration of neuraxial anesthesia for cesarean delivery, including single-shot spinal or combined spinal and epidural (CSE) anesthesia. Before initiating neuraxial anesthesia, 10 mg of metoclopramide was routinely administered to prevent intraoperative nausea and vomiting. For spinal anesthesia, a 27-gauge spinal needle (UNISIS Corp., Saitama, Japan) was inserted at the L2/3, L3/4, or L4/5 levels, as identified using ultrasound. For CSE anesthesia, an 18-gauge CSE Tuohy needle (B. Braun Aesculap Co. Ltd., Tokyo, Japan) was inserted at the same spinal level, followed by a needle-through-needle dural puncture using a 27-gauge spinal needle. A mixture of local anesthetic and opioids (12 mg hyperbaric bupivacaine, 10 µg fentanyl, and 0.15 mg preservative-free morphine) was administered intrathecally. For CSE anesthesia, a multi-orifice closed-end epidural catheter was inserted into the epidural space cephalad at 3–5 cm. A drug mixture of 2% lidocaine with 5 µg/mL epinephrine was administered as an epidural bolus, if applicable. A continuous infusion of oxytocin at a rate of 5–10 IU/h with 1–3 IU bolus was initiated after delivery. When the parturient experienced pain or discomfort after childbirth, systemic analgesics (intravenous fentanyl, morphine, meperidine, acetaminophen, and nitrous oxide), sedatives (intravenous diazepam, midazolam, droperidol, propofol, and sevoflurane), and antiemetics (intravenous metoclopramide, droperidol, and dexamethasone) were administered individually or in combination, at the discretion of the attending anesthesiologist.

### Postoperative management

Because we excluded emergencies, all the elective cesarean sections were completed during the daytime, and the patients were subsequently transferred to the ward. During the first 24 h after the surgery, the patients were administered 1 g of intravenous acetaminophen every 6 h at set intervals. For patients weighing less than 50 kg, the dose was adjusted to 600 mg per administration. Pain intensity was assessed using a 0–10 numeric rating scale (NRS), and if a patient requested it, a rescue analgesic of 15 mg pentazocine or 0.15 mg buprenorphine was administered intravenously. Respiratory monitoring was performed hourly by midwives 24 h after the administration of neuraxial morphine. The respiratory rate was measured using an ECG module, allowing midwives to record it remotely without disturbing the patient. However, they visited the bedside for other reasons, such as blood pressure measurements and assisting with breast milk expression. All bedside visits during the first night after surgery were documented in the patients’ medical records. The day after the surgery, the obstetric anesthesia team visited the patient, recorded the patient’s pain level using the NRS, and noted any postoperative nausea and vomiting, itchiness, sleep disturbances, or other complications related to anesthesia. Nausea and itchiness were recorded using four grades: none, mild (no medication required), moderate (improved with medication), and severe (did not improve with medication) [6]. The urinary catheter, which was inserted during the surgery, was removed the morning after the surgery.

### One-month postpartum check-up

At the 1-month postpartum clinic visit, the midwives assessed and recorded the EPDS, mother-infant bonding scale, and Childcare Support Checklist using the paper-based questionnaire screening sheets. The Japanese version of the mother-infant bonding scale (MIBS-J) is a simple self-report questionnaire designed to detect problems with a mother’s feelings towards her newborn baby [7]. The Childcare Support Checklist is an assessment tool for support from the husband, surroundings, and other environmental, financial, or social factors related to childrearing (Supplementary material [8]). The presence of economic poverty was assessed using a Childcare Support Checklist at 1 month postpartum. Generally, the MIBS-J and Childcare Support Checklists are often used 1 month postpartum along with the EPDS in Japan.

### Statistical analysis

The sample size for this study was designed based on feasibility considerations owing to the lack of established data on the relationship between acute postpartum sleep disorders and PPD. Postpartum EPDS scores between the Poor and Good Sleep groups were compared. Other factors, such as maternal background and intraoperative, neonatal, and postoperative information, were also compared between the two groups. All statistical analyses were performed using the SAS statistical software package, version 9.4 (SAS Institute, Cary, NC, USA). Normally distributed continuous variables are expressed as mean ± standard deviation, and non-normally distributed variables are expressed as median and interquartile range. Categorical data are expressed as absolute numbers and percentages. Continuous variables were compared using the Student’s t-test or Mann–Whitney U test, as appropriate. Categorical variables were compared using Pearson’s chi-squared or Fisher’s exact tests. Propensity scores were calculated using multivariate logistic regression, with six potential confounders: parity, age, body mass index (BMI), psychiatric disorders, postoperative NRS, and economic poverty. A 1:2 propensity score matching analysis without replacement (greedy matching algorithm) with a caliper width of 0.2 of the standard deviation of the logit of the propensity score was conducted. After propensity score matching, the outcomes of continuous variables were compared using the Student’s t-test or Mann–Whitney U-test, as appropriate. Categorical variables were compared using Pearson’s chi-squared or Fisher’s exact tests. Statistical significance was set at *P* < 0.05.

## Results

**Fig. 1:**
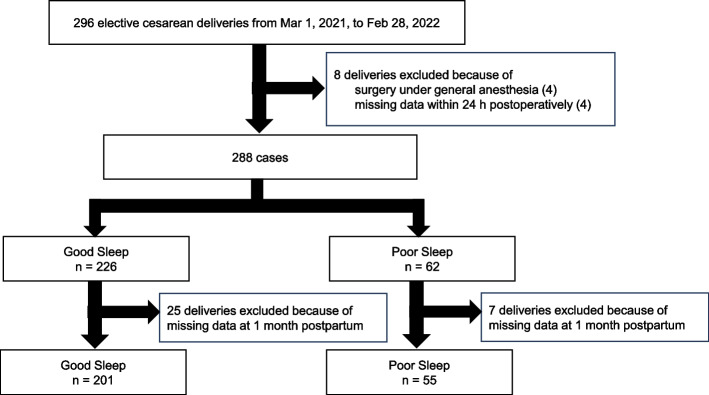
Flow diagram. Of the 296 patients that met the inclusion criteria, 8 cases were excluded from the analysis owing to surgery under general anesthesia (*n* = 4) or missing data within the first 24 h postoperatively (*n* = 4). Among the 288 patients who had complete data 24 h postoperatively, 32 were lost to follow-up at 1 month postpartum, (7 and 25 in the Poor and Good Sleep groups, respectively). Finally, 256 patients (55 and 201 in the Poor and Good Sleep groups, respectively) were included for further analysis

During the study period, 296 cases met the inclusion criteria. Of these, 8 cases were excluded from the analysis owing to surgery under general anesthesia (n = 4) or missing data within the first 24 h postoperatively (*n* = 4). Among the 288 patients who had complete data 24 h postoperatively, 32 were lost to follow-up at 1 month postpartum, (7 and 25 in the Poor and Good Sleep groups, respectively). Finally, 256 cases were included for analysis, with 55 in the Poor Sleep group and 201 in the Good Sleep group (Fig. 1).

Table 1 summarizes the maternal characteristics, including crude and propensity score-matched data. In the crude data, maternal BMI at delivery was significantly lower in the Poor Sleep group than in the Good Sleep group (24.6 ± 3.2 vs. 26.0 ± 4.7, *P* = 0.031). Moreover, maternal psychological and endocrine disorders and multiple gestations were more prevalent in the Poor Sleep group than in the Good Sleep group (12.7% vs. 4.5%, *P* = 0.025; 18.2% vs. 4.0%, *P* = 0.0003; and 38.2% vs. 17.9%, *P* = 0.001, respectively). Maternal endocrine disorders and multiple gestations were more prevalent in the Poor Sleep group, even after propensity score matching (16.7% vs. 3.9%, *P* = 0.006 and 38.9% vs. 18.4%, *P* = 0.005, respectively). Most endocrine disorders were subclinical or overt hypothyroidism. In addition, only after the propensity score matching, preoperative hypnotic drug use was significantly more prevalent in the Poor Sleep group compared with the Good Sleep group (3.7% vs. 0.0%, *P* = 0.0049).

**Table 1:** Patient characteristics

	Crude				Propensity score matching			
	Poor sleep *n* = 55	Good sleep *n* = 201	*P*	Std diff	Poor sleep *n* = 54	Good sleep *n* = 103	*P*	Std diff
Age, year (SD)	34.8 (5.0)	33.8 (4.9)	0.209	0.202	34.8 (5.0)	34.6 (5.1)	0.744	0.197
Height, cm (SD)	158.5 (5.8)	157.8 (5.6)	0.391	0.123	158.7 (5.7)	157.5 (5.6)	0.214	0.212
Weight, kg (SD)	61.9 (9.2)	64.9 (12.3)	0.093	−0.276	62.1 (9.2)	60.7 (9.0)	0.359	0.155
BMI, kg/m^2^	24.6 (3.2)	26.0 (4.7)	0.031	−0.348	24.6 (3.3)	24.4 (3.2)	0.726	0.062
Multipara, n (%)	31 (56.4)	129 (64.2)	0.289	−0.16	30 (55.6)	63 (61.2)	0.497	−0.114
Hospitalization days before delivery, days (IQR)	1 (1, 33)	1 (1, 13)	0.093	0.24	1.0 (1.0, 30)	1.0 (1.0, 25.0)	0.433	0.128
Preoperative Hb, g/dL (SD)	11.2 (0.9)	11.2 (1.0)	0.92	0	11.2 (0.9)	11.2 (1.0)	0.96	0
HDP, n (%)	3 (5.5)	19 (9.5)	0.349	−0.152	3 (5.6)	6 (5.8)	0.945	−0.009
DM, n (%)	5 (9.1)	24 (11.9)	0.555	−0.914	5 (9.3)	8 (7.8)	0.747	
Cardiac disease, n (%)	3 (5.5)	8 (4.0)	0.633	0.071	3 (5.6)	7 (6.8)	0.762	−0.05
Respiratory disease, n (%)	7 (12.7)	34 (16.9)	0.453	−0.118	6 (11.1)	19 (18.4)	0.233	−0.207
Renal disease, n (%)	2 (3.6)	4 (2.0)	0.475	0.097	2 (3.7)	2 (1.9)	0.506	0.109
Gastrointestinal disease, n (%)	7 (12.7)	26 (12.9)	0.968	−0.0006	7 (13.0)	17 (16.5)	0.558	−0.099
Hematologic disease, n (%)	0 (0.0)	3 (1.5)	0.362	−0.175	0 (0.0)	2 (1.9)	0.303	−0.197
Endocrine disease, n (%)	10 (18.2)	8 (4.0)	< 0.001	0.464	9 (16.7)	4 (3.9)	0.006	0.431
Neurologic disease, n (%)	2 (3.6)	5 (2.5)	0.643	0.064	2 (3.7)	2 (1.9)	0.508	0.109
Psychiatric disorder, n (%)	7 (12.7)	9 (4.5)	0.025	0.296	6 (11.1)	7 (6.8)	0.351	0.151
Psychiatric medication use, n (%)	4 (7.3)	5 (2.5)	0.088	0.224	3 (5.6)	2 (1.9)	0.221	0.196
Antidepressants, n (%)	4 (7.3)	5 (2.5)	0.088	0.224	3 (5.6)	2 (1.9)	0.221	0.196
Antipsychotic drugs, n (%)	0 (0.0)	2 (1.0)	0.458	−0.142	0 (0.0)	2 (1.9)	0.303	−0.197
Anti-anxiety drugs, n (%)	2 (3.6)	1 (0.5)	0.055	0.22	2 (3.7)	1 (1.0)	0.235	0.179
Hypnotics, n (%)	3 (5.5)	3 (1.5)	0.083	0.219	2 (3.7)	0 (0.0)	0.049	0.277
ASA-PS > 2, n (%)	0 (0.0)	9 (4.5)	0.11	−0.307	0 (0.0)	3 (2.9)	0.205	−0.307
Multiple gestation, n (%)	21 (38.2)	36 (17.9)	0.001	0.464	21 (38.9)	19 (18.4)	0.005	0.466
Indication for cesarean delivery, n (%)								
Breech	5 (9.1)	15 (7.5)	0.041	0.058	5 (9.3)	8 (7.8)	0.117	0.058
Multiple gestation	20 (36.4)	36 (17.9)		0.425	20 (37.0)	19 (18.4)		0.425
Placental position	9 (16.4)	35 (17.4)		−0.027	9 (16.7)	21 (20.4)		−0.027
Uterine scar including previous	19 (34.5)	100 (49.8)		−0.314	18 (33.3)	51 (49.5)		−0.314
cesarean Others	2 (3.6)	15 (7.5)		−0.171	2 (3.7)	4 (3.9)		−0.171

*BMI* body mass index, *HDP* hypertensive disorder of pregnancy, *DM* diabetes mellitus, ASA-PS American Society of Anesthesiologists physical status classification system, *SD* standard deviation, *IQR* interquartile range, *Std diff* standardized difference, *Hb* hemoglobin

Table 2 shows the intraoperative, postoperative, and neonatal data, including the crude and propensity score-matched data. In both datasets, no significant differences were observed in intraoperative anesthesia management between the two groups. Postoperatively, the highest pain score measured with the NRS, incidence of NRS > 3, and the requirement for rescue opioid analgesia were higher in the Poor Sleep group compared with the Good Sleep group in the crude data. After propensity score matching, no significant differences were observed between the two groups. Neonatal outcomes were similar in the crude and propensity-matched data.

**Table 2:** Intraoperative management, postoperative management, and neonatal outcome

	Crude				Propensity score matching			
	Poor sleep *n* = 55	Good sleep *n* = 201	*P*	Std diff	Poor sleep *n* = 54	Good sleep *n* = 103	*P*	Std diff
Neuraxial anesthesia, *n* (%)								
CSE	16 (29.1)	66 (32.8)	0.598	−0.08	16 (29.6)	38 (36.9)	0.363	0.155
Spinal	39 (70.9)	136 (67.2)		0.08	38 (70.4)	65 (63.1)		−0.155
Sedatives, *n* (%)	10 (18.2)	36 (17.9)	0.963	0.008	10 (18.5)	21 (20.4)	0.78	−0.048
Rescue analgesics, *n* (%)	6 (10.9)	34 (16.9)	0.277	−0.174	5 (9.3)	17 (16.5)	0.214	−0.216
Antiemetics								
Metoclopramide	53 (96.4)	199 (99.0)	0.162	−0.174	52 (96.3)	101 (98.1)	0.506	0.109
Dexamethasone	10 (18.2)	48 (23.9)	0.371	−0.14	10 (18.5)	25 (24.3)	0.411	0.141
Droperidol	0 (0.0)	0 (0.0)			0 (0.0)	0 (0.0)		
Blood loss, g (SD)	1 364 (733)	1 213 (551)	0.097	0.233	2 374 (736)	2 286 (563)	0.405	0.134
Transfusion, *n* (%)	4 (7.3)	5 (2.5)	0.088	0.224	4 (7.4)	4 (3.9)	0.34	0.152
Maximum pain NRS ≤ 24 h (SD)	5.5 (1.8)	4.8 (1.8)	0.018	0.386	5.5 (1.9)	5.1 (1.6)	0.198	0.232
NRS > 3, *n* (%)	48 (87.3)	149 (74.1)	0.04	0.339	47 (87.0)	90 (87.4)	0.951	−0.012
NRS > 5, *n* (%)	22 (40.0)	64 (31.8)	0.256	0.172	22 (40.7)	36 (35.0)	0.475	0.246
Opioid rescue, *n* (%)	18 (32.7)	28 (13.9)	0.001	0.456	18 (33.3)	21 (20.4)	0.075	0.294
Opioid dose ≤ 24 h (morphine equivalent), mg (SD)	8.3 (4.85)	6.1 (2.47)	0.043	0.572	8.3 (4.85)	6.1 (2.63)	0.082	0.564
PONV, *n* (%)								
None	36 (65.5)	147 (73.1)		−0.165	35 (64.8)	73 (70.9)		−0.131
Mild	10 (18.2)	29 (14.4)	0.16	0.103	10 (18.5)	17 (16.5)	0.361	0.053
Moderate	6 (10.9)	23 (11.4)		−0.016	6 (11.1)	12 (11.7)		−0.019
Severe	3 (5.5)	2 (1.0)		0.256	3 (5.6)	1 (1.0)		0.26
Pruritus, *n* (%)								
None	19 (34.5)	100 (49.8)		−0.314	19 (35.2)	53 (51.5)		−0.333
Mild	23 (41.8)	67 (33.3)	0.247	0.176	22 (40.7)	32 (31.1)	0.284	0.201
Moderate	11 (20.0)	28 (13.9)		0.163	11 (20.4)	15 (14.6)		0.153
Severe	2 (3.6)	6 (3.0)		0.034	2 (3.7)	3 (2.9)		0.045
Narcotic medication, *n* (%)	10 (18.2)	37 (18.4)	0.969	−0.005	10 (18.5)	22 (21.4)	0.675	−0.073
Postoperative Hb, g/dL (SD)	10.6 (1.52)	10.5 (1.31)	0.53	0.07	10.6 (1.5)	10.4 (1.4)	0.468	0.068
Nurse visit during night, times (SD)	5.0 (1.5)	5.4 (1.4)	0.089	−0.005	5.1 (1.6)	5.3 (1.4)	0.246	0.144
	Poor sleep *n* = 76	Good sleep *n* = 240	*P*	Std diff	Poor sleep *n* = 75	Good sleep *n* = 125	*P*	Std diff
Birth weight, g (SD)	2 546.3 (366.8)	2 649.0 (424.3)	0.059	−0.486	2 553.7 (363.4)	2 580.7 (399.6)	0.633	−0.07
Apgar score at 1 min < 7, *n* (%)	3 (3.9)	18 (7.5)	0.279	−0.156	3 (4.0)	10 (8.0)	0.267	−0.169
Apgar score at 5 min < 7, *n* (%)	0 (0.0)	7 (2.9)	0.132	−0.244	0 (0.0)	3 (2.4)	0.176	−0.222
UA pH, (SD)	7.33 (0.03)	7.33 (0.03)	0.316	0	7.33 (0.03)	7.33 (0.03)	0.572	0
NICU admission, *n* (%)	28 (36.8)	62 (25.8)	0.064	0.239	27 (36.0)	36 (28.8)	0.289	−0.063
NICU stay, days (IQR)	0 (0, 5)	0 (0, 1)	0.122	0.152	0 (0, 5)	0 (0, 3)	0.372	0.115

*CSE* combined spinal-epidural, *NRS* numeric rating scale, *PONV* postoperative nausea and vomiting, *UA* umbilical artery, *NICU* neonatal intensive care unit, *SD* standard deviation, *IQR* interquartile range, *Std diff* standardized difference, *Hb* hemoglobin

Table 3 shows the crude and propensity-matched data for the 1-month postpartum EPDS scores. In both analyses, the EPDS total score was significantly higher in the Poor Sleep group than in the Good Sleep group (6.7 ± 5.2 vs. 4.5 ± 4.3, *P* = 0.007, after propensity score matching). The maternal risk for PPD (i.e., EPDS ≥ 9) was also significantly higher in the Poor Sleep group (31.5% vs. 17.5%, *P* = 0.045, odds ratio 2.17, 95% confidential interval 1.01 to 4.67, after propensity score matching) than in the Good Sleep group. Other 1-month postpartum confounders, including the total MIBS-J score and presence of economic poverty assessed using the Childcare Support Checklist, were similar between the two groups.

**Table 3:** One-month postpartum outcomes

	Crude			Propensity score matching		
	Poor sleep *n* = 55	Good sleep *n* = 201	*P*	Poor sleep *n* = 54	Good sleep *n* = 103	*P*
EPDS total score, mean (SD)	6.6 (5.2)	4.0 (3.9)	<.0001	6.7 (5.2)	4.5 (4.3)	0.007
EPDS total score, median (IQR)	7.0 (3.0, 9.0)	3.0 (1.0, 6.0)		7.0 (3.0, 9.0)	3.0 (1.0, 6.0)	
EPDS ≥ 9, *n* (%)	17 (30.9)	26 (12.9)	0.002	17 (31.5)	18 (17.5)	0.045
EPDS ≥ 13, *n* (%)	5 (9.1)	6 (3.0)	0.045	5 (9.3)	5 (4.9)	0.283
EPDS score by items, (SD)						
1	0.2 (0.5)	0.1 (0.4)	0.227	0.2 (0.5)	0.1 (0.4)	0.367
2	0.3 (0.6)	0.1 (0.4)	0.011	0.3 (0.6)	0.2 (0.5)	0.088
3	1.3 (1.1)	0.9 (0.9)	0.002	1.3 (1.1)	1.0 (0.9)	0.06
4	1.1 (1.0)	0.7 (0.9	0.001	1.1 (1.0)	0.8 (0.9)	0.033
5	0.7 (0.9)	0.3 (0.6)	0.00002	0.7 (0.9)	0.4 (0.7)	0.006
6	1.4 (0.7)	1.2 (0.8)	0.02	1.4 (0.7)	1.2 (0.8)	0.047
7	0.3 (0.6)	0.2 (0.5)	0.038	0.3 (0.6)	0.2 (0.6)	0.277
8	0.7 (0.8)	0.4 (0.7)	0.003	0.7 (0.8)	0.5 (0.7)	0.041
9	0.4 (0.7)	0.1 (0.4)	< 0.0001	0.4 (0.7)	0.1 (0.4)	0.003
10	0.1 (0.4)	0.1 (0.3)	0.145	0.1 (0.4)	0.1 (0.3)	0.291
MIB-J total score, (SD)	1.9 (1.7)	1.5 (1.8)	0.132	1.9 (1.7)	1.7 (1.9)	0.461
Economic poverty, *n* (%)	9 (16.4)	29 (14.4)	0.721	9 (16.7)	17 (16.5)	0.979

*EPDS* Edinburgh Postnatal Depression Scale, *MIB-J* Japanese version of mother-infant bonding scale, *SD* standard deviation

## Discussion

This study revealed an association between self-reported poor sleep quality during the first day after cesarean delivery and the EPDS score 1 month after childbirth. This association remained even after considering confounding factors, such as age, BMI, parity, psychiatric disorders, and postoperative NRS. Therefore, disturbed sleep immediately after cesarean delivery can be considered an independent risk factor for PPD.

These findings are consistent with those of previous studies. A systematic review of 13 observational studies found a significant relationship between sleep disturbance and PPD, with effect sizes ranging from moderate to large and very large (0.6–1.7) [3]. However, previous evidence had limitations, as most researchers did not specify the confounding factors that were controlled [3]. In this study, we conducted propensity score matching to account for the potential confounders of PPD. We included covariates previously reported to be associated with PPD, such as age, BMI, parity, psychiatric disorders, and economic poverty [1, 2, 9–11]. Our findings suggest that self-reported poor sleep quality immediately after cesarean delivery can independently associated with the risk of PPD.

Our findings also suggest that maternal endocrine diseases, predominantly thyroid disorders, and multiple gestations are associated with self-reported poor sleep quality in the immediate postpartum period. These factors are potentially linked to known contributors to sleep disturbances, such as thyroid dysfunction-induced insomnia, obstructive sleep apnea, and restless legs syndrome [12], as well as the demands of neonatal care and fatigue [13]. In addition, our results indicate that antenatal hypnotics are associated with self-reported poor sleep quality during acute postpartum period, suggesting that mothers experiencing sleep disturbances before delivery are at a higher risk of encountering sleep disturbances after delivery.

Our study focused on sleep disturbances during the first night of postpartum hospitalization as a potential risk factor for PPD. The strength of our study methodology lies in the rigorous standardization of the sleeping environment for all participants, as they were uniformly situated in the same hospital setting and separated from their newborns. Prior research has highlighted that acute sleep disturbances during postpartum hospitalization can be influenced by various factors unique to the institutional environment, with newborn care being the primary contributing factor [13]. In addition, disruptions due to nurse visits for procedures such as vital sign assessments and the need for bathroom visits have been reported [13]. Our study uniquely evaluated patient sleep quality in a standardized hospital setting. This environment encompassed consistent bedside arrangements, including monitoring equipment, similar frequencies of nurse visits, and the utilization of urinary catheterization during the first night postoperatively. Furthermore, to minimize the potential influence of postoperative pain on sleep disturbance, we conducted a propensity score matching with postoperative pain as a covariate. By implementing these measures, we aimed to enhance the internal validity of our findings and provide a more accurate assessment of the relationship between sleep disturbance and PPD. On the other hand, because these conditions are specific to our institution, the generalizability of our findings to other clinical settings may be limited.

Our study had several limitations. First, this was a retrospective observational study, and although we performed propensity score matching with maternal backgrounds, participant characteristics were not necessarily balanced. For example, a recent meta-analysis suggested that socioeconomic factors, including marital status, educational level, social support, financial problems, and substance abuse, may impact the development of PPD [1]. Although we used the Childcare Support Checklist to screen for socioeconomic status, future studies should consider conducting more detailed evaluations of socioeconomic risk factors. Furthermore, even after propensity score matching, endocrine disorders and multiple gestations remained more prevalent in the poor sleep quality group, suggesting residual imbalance. A larger sample size in future studies may improve the precision of matching and allow for more robust control of potential confounders. Second, our study did not assess sleep disturbances using systematic measurement tools but only used a question about whether the participants had good or poor sleep to evaluate the sleep quality. While this method is straightforward and non-invasive, suitable for initial screening, it cannot capture detailed information about the specific quality and quantity of the patients’ sleep. Such details are important for identifying the causes of sleep disturbances and formulating targeted interventions. Previous studies have often used subjective measures, such as the General Sleep Disturbance Scale and Bergen Insomnia Scale, to assess sleep disturbances [3, 14]. Furthermore, in addition to self-reported measures, objective instruments such as polysomnography and actigraphy may be necessary to provide more precise assessments [15]. Third, we used the EPDS as a screening tool to identify women at risk of developing PPD because it is the most commonly used^1^; a definitive diagnosis of PPD requires psychiatric examination. A score of ≥ 9 on the EPDS indicates a high possibility of depression in the Japanese population [5]; however, a score of ≤ 8 does not rule out depression, and other mental disorders may also produce high scores. We did not analyze the actual incidence of PPD due to the failure to follow up with all women who had high postpartum EPDS scores. It is necessary to improve perinatal care practices to ensure follow-up and provide adequate interventions for those with high EPDS scores. Further prospective studies are required to overcome these limitations.

In conclusion, the results of this study showed a correlation between self-reported poor sleep quality within the first night after cesarean delivery and EPDS score 1 month after childbirth. Sleep disturbance on the day of delivery may be a risk factor for the development of PPD. This finding suggests a potential opportunity for early interventions to mitigate the risk of PPD, such as providing increased mental and social support and more intensive postpartum monitoring. Future research can explore the effectiveness of targeted interventions aimed at improving sleep quality in the immediate postpartum period to reduce the incidence of PPD.

## Supplementary Information

Supplementary Material 1.

## Data Availability

The data that support the findings of this study are available from the corresponding author, Yusuke Mazda.

## Abbreviations

PPD: Postpartum depression
EPDS: Edinburgh postnatal depression scale
CSE: Combined spinal and epidural
NRS: Numeric rating scale
MIBS-J: Japanese version of the mother-infant bonding scale
BMI: Body mass index
