# Supplementary material for: Self-reported poor sleep quality on the day of delivery is a potential risk factor for postpartum depression after cesarean delivery: a retrospective cohort study
Source: JA Clin Rep. 2025 Dec 29;11:70. doi: 10.1186/s40981-025-00833-5 (PMC12748453; doi:10.1186/s40981-025-00833-5)
Supplement: Supplementary file 1 — Supplementary Material 1. [file 40981_2025_833_MOESM1_ESM.docx]

**Supplementary material**

Questionnaire screening sheet of the Childcare Support Checklist

The author translated the Childcare Support Checklist from the Japanese material into English [8]. This checklist is an assessment tool to evaluate various environmental, financial, and social factors associated with child-rearing. It comprises nine items:

1. Abnormalities encountered during pregnancy.

2. Experience of miscarriage or stillbirth, serving as a screening for stressful events.

3. Presence of mental illness, assessing the risk of postpartum depression.

4. Support received from spouse, biological mother, or others during challenging times.

5. Financial worries.

6. Description of the current living environment.

7. Recent experience of a family member or close friend’s death, screening for recent stressful life events.

8. Confidence in understanding the reasons behind the baby’s crying. A negative answer indicates a potential risk for neglect.

9. Presence of thoughts about harming the baby, indicating a potential risk for child abuse.

A positive response to item 5 indicates potential economic hardship. The outcomes of item 5 were incorporated as a confounding variable of economic poverty in propensity score matching.
